# Extracellular Vesicle-Based Strategies for Tumor Immunotherapy

**DOI:** 10.3390/pharmaceutics17020257

**Published:** 2025-02-14

**Authors:** Luksika Jiramonai, Xing-Jie Liang, Mengliang Zhu

**Affiliations:** 1 Chinese Academy of Sciences (CAS), Key Laboratory for Biomedical Effects of Nanomaterials and Nanosafety, CAS Center for Excellence in Nanoscience, National Center for Nanoscience and Technology of China, Beijing 100190, China; luksika2022@nanoctr.cn; 2 University of Chinese Academy of Sciences, Beijing 100049, China

**Keywords:** immunotherapy, extracellular vesicles, tumor microenvironment, immune evasion, immune checkpoint inhibitors

## Abstract

Immunotherapy is one of the most promising approaches for cancer management, as it utilizes the intrinsic immune response to target cancer cells. Normally, the human body uses its immune system as a defense mechanism to detect and eliminate foreign objects, including cancer cells. However, cancers develop a ‘switch off’ mechanism, known as immune checkpoint proteins, to evade immune surveillance and suppress immune activation. Therefore, significant efforts have been made to develop the strategies for stimulating immune responses against cancers. Among these, the use of extracellular vesicles (EVs) to enhance the anti-tumor immune response has emerged as a particularly promising approach in cancer management. EVs possess several unique properties that elevate the potency in modulating immune responses. This review article provides a comprehensive overview of recent advances in this field, focusing on the strategic usage of EVs to overcome tumor-induced immune tolerance. We discuss the biogenesis and characteristics of EVs, as well as their potential applications in medical contexts. The immune mechanisms within the tumor microenvironment and the strategies employed by cancers to evade immune detection are explored. The roles of EVs in regulating the tumor microenvironment and enhancing immune responses for immunotherapy are also highlighted. Additionally, this article addresses the challenges and future directions for the development of EV-based nanomedicine approaches, aiming to improve cancer immunotherapy outcomes with greater precision and efficacy while minimizing off-target effects.

## 1. Introduction

Research on tumor immunotherapy has been expanding extensively due to many intrinsic advantages. The satisfactory efficacies and outcomes primarily result from the activation of the body’s immune system to combat cancer. Normally, the body has its own mechanism to detect antigens and eliminate foreign substances, including abnormal cells like cancer cells. The immune system also utilizes immune checkpoint mechanisms to regulate and maintain immunity levels, preventing an overactive immune response and protecting healthy cells.

Tumors take advantage of immune checkpoint mechanisms and develop their components to mimic these processes. This allows tumors to grow and survive without detection by immune cells. Over the past several decades, advancements in immunology have enabled the development of several immunotherapeutic strategies, including checkpoint inhibitors, chimeric antigen receptor (CAR) T-cell therapy, cancer vaccines, and monoclonal antibodies. Nowadays, there are several immunotherapeutic treatments approved by the U.S. Food and Drug Administration (FDA). The enhancement of the immune response provides an alternative approach to cancer treatment, supplementing traditional chemotherapy, which directly targets cancer cells but has great toxic and other side effects. These immunotherapeutic approaches hold great promise for tumor management due to their ability to induce durable responses by strengthening the immune system rather than directly targeting malignant cells [1]. Despite some successes, immunotherapy is not universally effective. Upon the activation of immune cells, immunotherapies may accidentally stimulate immune cells to attack healthy cells, causing undesirable side effects. Therefore, ongoing researches are still needed to understand the complex mechanisms [2].

Many early immunotherapeutic strategies, including traditional approaches like immune checkpoint inhibitors (ICIs), have encountered these challenges. One of the major challenges of tumor immunotherapy is the development of resistance by cancer cells, which continuously emerge as part of their survival strategy. Immunotherapy tolerance occurs when the immune system exhibits a reduced response to the administered immunotherapeutic approaches, eventually leading to treatment failure. This tolerance also allows tumors to evade immune detection, promoting growth, metastasis, and relapse following treatment [3].

Extracellular vesicles (EVs) have gained attention as a potential solution to various challenges in cancer treatment. Researchers have been working to manipulate the properties of naturally-derived EVs to enhance immunotherapeutic efficacy, re-induce immune responses, and ultimately overcome the tolerance exhibited by cancer cells [4]. EVs have emerged as a promising tool in the field of cancer treatment due to their unique characteristics. These nano-sized vesicles are naturally released by cells and are crucially involved in various biological processes [4,5]. Their intrinsic properties, such as low toxicity and high biocompatibility, make them advantageous as they originate from the body’s own cells. Additionally, diverse modifications can be applied to EVs to develop desired properties for individual needs [4]. In this review, we summarize the biogenesis and classification of EVs, their clinical applications, and potential modifications for medical use. The roles of EVs in tumor immunotherapy are specifically highlighted as alternative therapeutic tools in tumor immunotherapy for minimizing the deleterious effects that may possibly occur at the off-target sites. Even though there are advantages to using EVs in tumor immunotherapy, there are some aspects that need to be focused on. These challenges and limitations are also discussed in order to improve the development of these EV-based nanomedicines. Further directions for EV-based nanomedicines to overcome the immunosuppressive conditions associated with tumors are also discussed.

## 2. The Biogenesis and Characteristics of Extracellular Vesicles

### 2.1. Classification of Extracellular Vesicles

EVs are cell-derived vesicles that are composed of a phospholipid bilayer as a major component [6]. The history of EVs dates back to 1945, when a researcher obtained the first glimpse of their existence after using high-speed separation techniques and fixing a blood clot within a shorter time than previously performed [7]. Then, a year later, in 1946, ‘particulate fraction’ was discovered when high-speed centrifugation force was applied [8]. In 1967, the first electron microscopic images of these particles were captured, and they were characterized as ‘platelet dust’ [9]. Even after a long period of knowing, the term ‘extracellular vesicles’ was first used many years later in the title of a scientific article published in 1971, in which the biogenesis of EVs from algae was detected using electron microscopy [10]. According to the recent Minimal Information for Studies of Extracellular Vesicles (MISEV) 2023, the term ‘extracellular vesicles’ refers to lipid bilayer nano-sized particles that are naturally secreted by all cells and do not self-replicate [11]. Contents in the EVs can vary widely, ranging from lipids, proteins, and many genomic contents, including deoxyribonucleic acid (DNA) and Ribonucleic Acid (RNA) [12].

**Figure 1:**
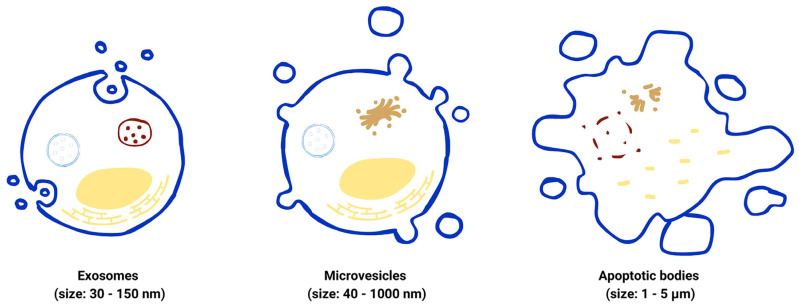
Classification and biogenesis of extracellular vesicles. Three subtypes of extracellular vesicles: exosomes, microvesicles, and apoptotic bodies.

The categorization systems for EVs are diverse. The guideline for rough categorization is to classify EVs by their size, small EVs for the ones that are less than 200 nm and large EVs for the ones exceeding 200 nm [13]. Recent studies indicate that, when considering size, biogenesis, and release pathways, EVs can be divided into three main subpopulations: exosomes, microvesicles or microparticles, and apoptotic bodies (Figure 1). These three subtypes are collectively referred to as classical EVs [14]. Apart from these three main EVs, other nomenclatures, with different classifications according to their organ-specific biogenesis, are also used to identify some EVs [14]. However, most researches usually refer to these three subtypes, and their corresponding original sources and specific markers are listed in Table 1.

Exosomes, a subset of EVs, were first mentioned in 1981 [15]. Exosomes or intraluminal vesicles (ILVs), are the smallest of all three EV subtypes, with sizes ranging from 30–150 nm [12]. Their biogenesis process generally involves three main steps: generation, transportation, and release. The process starts from the formation of endosomes from the cellular surface until endosomes are formed in the early and late stages, respectively. Then, ILVs are formed inside the multivesicular endosomes (MVEs) and are later released out of the cells via the exocytosis process, in which the MVEs fuse with the plasma membrane [14]. The main contents of exosomes include nucleic acids, proteins, and lipids. Exosomes, generally found in bodily fluids, are essential for various biological processes, especially intercellular communication, exchanging the biological signaling molecules between cells [16]. The membrane of exosomes contains specific tetraspanin proteins, such as cluster of differentiation (CD) 9, CD81, and CD63, which can be used as biomarkers to distinguish exosomes from other subpopulations of EVs [6].

Microvesicles, or microparticles, originate via the budding process of the plasma membrane, resulting in a size ranging from 40 to 1000 nm [14]. The contents of microvesicles, similar to those of other EV subpopulations, also carry various proteins, genetic materials, and other biological molecules. These exfoliated vesicles exhibit some enzymatic activities that may be related to physiological functions [15]. The producing and up-taking processes of microvesicles significantly depend on the physiological microenvironment [12]. According to the biogenesis of microvesicles, their protein contents mainly relate to cytoplasm and the plasma membrane. Apart from cytosolic and plasma membrane proteins, other proteins found in microvesicles include heat shock proteins and cytoskeletal proteins. The functions of microvesicles, similar to those of exosomes, involve communication between cells. Aside from the difference in size between microvesicles and exosomes, there are no specific biomarkers to distinguish microvesicles [12].

Apoptotic bodies, also known as apoptosomes, are the largest type of EVs, with a size range of 1–5 µm. They are formed from dying cells during the apoptotic process, resulting from cellular contraction and higher levels of hydrostatic pressure. Therefore, apoptotic bodies function as a part of a clearance pathway to clear out the unwanted substances [17]. The contents of apoptotic bodies differ from those of exosomes and microvesicles. The main components of apoptotic bodies may include cellular organelles, DNA, and some chromatin [12,17]. Regarding the isolation method, unlike exosomes and microvesicles in which ultracentrifugation is useful, there is no specific isolation method for apoptotic bodies [17].

Apart from the classic categorization of EVs, biogenesis process, original source, and size are also used for classification. In terms of size, a size of 200 nm is used as criteria to divide EVs into small and large EVs [13]. Other specific categorizations include oncosomes (from cancer cells), matrix vesicles (from extracellular matrix), autophagic EVs (secreted from of amphisomes, resulting from the fusion of autophagosomes and endosomes), stress EVs or stressosomes (secreted from stress-induced cells), and prostasomes (secreted from prostate gland) [14,18]. The most recent category of EVs is migrasomes, which are the specialized EVs produced during migracytosis, a mechanism of cell migration [19].

### 2.2. Isolation Methods for Extracellular Vesicles

The potential applications of EVs, particularly in nanomedicine, have prompted numerous efforts to isolate EVs, specifically exosomes from bodily fluids. Ultracentrifugation-based methods have been used for isolation since the establishment of this technique, as they exploit the unique size characteristics of each EV subtype. Ultracentrifugation is also considered the current gold standard for EV isolation. This method involves a series of centrifugation steps, beginning with lower forces to remove unwanted cell debris and larger vesicles, and progressing to high speeds to obtain exosome pellets [12]. The ultracentrifugation technique is widely used due to its affordability and lack of requirement for specialized equipment, accounting for approximately 81% of all available isolation methods [20]. The development of isolation techniques has expanded to include various ultracentrifugation-based approaches, such as differential centrifugation and rate-zonal centrifugation [12].

Besides ultracentrifugation, the principle of size differentiation in EVs is also applied in ultrafiltration, where suspensions containing EVs are passed through specific size exclusion membranes [21]. However, the drawback of this method is the contamination of EVs with other biomaterials of similar sizes. To address this issue, ultracentrifugation and ultrafiltration techniques are often combined [22].

Apart from the size-based methods, biochemical properties are used for isolating processes. Approaches based on immunoaffinity rely on the interaction between the surface antigens of EVs and specific antibodies. Examples of these approaches are Enzyme-Linked Immunosorbent Assay (ELISA) and immunoprecipitation [23]. Due to their specificity, these techniques can be applied for further isolations, such as separating specific subtypes of EVs from the overall EV pool [24]. However, further development for EV isolation methods is still necessary, as the biological complexity, including cell debris and other biological components, poses significant challenges that potentially hinder current isolation techniques [12].

### 2.3. Identification and Analysis of Extracellular Vesicles

The identification step is crucial after the isolation of EVs. Generally, the analysis can be categorized into two major types, focusing on either physical or biochemical aspects. Physical analysis mainly elucidates the size and concentration of the vesicles. Analysis methods include nanoparticle tracking analysis (NTA), dynamic light scattering (DLS), and electron microscopy. NTA and DLS provide similar information, such as the size and concentration of the vesicles, as both are based on the Brownian motion of particles. However, NTA is more suitable for the heterogenous samples, while DLS requires a smaller sample size [25]. The morphology and size distribution of vesicles can be examined using transmission electron microscopy (TEM) and scanning electron microscopy (SEM). These microscopy techniques differ in how electrons interact with and are detected from the samples. Although the results from these two microscopy methods are similar in terms of size distribution, the morphologies appear different due to their distinct sample preparation processes [12].

Another analysis is based on the biochemical compositions of EVs, including immunodetection methods. These techniques utilize the specific antigens on the vesicle surface as marker proteins, which are recognized by antibodies. Each EV subtype has its own unique specific markers, with tetraspanins (CD9, CD63, CD81) for exosomes [26], ADP-ribosylation factor 6 (ARF6) and β1 integrin for microvesicles [27,28], and Annexin V for apoptotic bodies [29]. These analyses are often used to assess the purity of vesicles following isolation. Common methods associated with this concept include Western blotting and flow cytometry [12]. Western blotting involves several steps, starting from the lysis of harvested samples containing EVs, followed by the denaturation of samples, and the separation by Sodium Dodecyl Sulfate Polyacrylamide Gel Electrophoresis (SDS-PAGE) according to their size. Then, those proteins are transferred to a membrane and exposed to specific antibodies to detect the presence of antigens [30]. Flow cytometry also utilizes the principle of immunodetection, utilizing fluorescent antibodies to identify EV markers. This technique provides information on the size, concentration, and subpopulations of EVs. However, there are some limitations, including the requirement of advanced equipment, the detection limit for EVs with a size less than 100 nm, and challenges in detecting aggregated EVs [31,32].

## 3. Extracellular Vesicles for Diagnostic and Therapeutic Potentials

**Figure 2:**
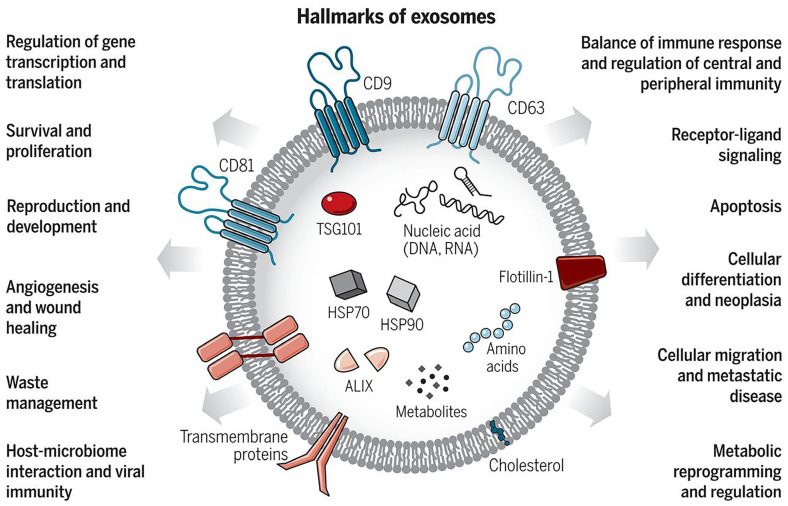
The significant compositions of exosomes with cellular functions. Exosomes, a subtype of extracellular vesicles, are produced by all cell types in the body and function as intercellular communication molecules. These vesicles can travel far from their cells of origin and may reflect the status of the originating cells. Reproduced with permission from Raghu Kalluri, Valerie S. LeBleu, Science; published by The American Association for the Advancement of Science, 2020 [16].

Extracellular vesicles play crucial roles in various biological processes, including cell–cell communications and the transfer of biological molecules. The overview list of significant functions is also shown in Figure 2 [16]. Apart from the normal functions of EVs as cellular messengers, they also have a significant impact on clinical aspects. Changes in body function contribute to the secretion of a mixture of various EV subtypes containing different compositions, compared to those from the normal status. The internal contents in EVs also correlate with the content of their origin.

### 3.1. Extracellular Vesicles as Diagnostic Tool

Given their biological functions, EVs may play significant roles in various clinical applications. One key aspect is the use of EVs as a non-invasive diagnostic tool, which is advantageous due to their natural secretion as part of normal physiological processes. EVs can be isolated from bodily fluids using various techniques, with ultracentrifugation being the most common and conventional method. However, this centrifuge-based approach has drawbacks, including being time-consuming and its susceptibility to degradation, leading to the exploration of alternative isolation methods.

Previous research on an endometrial cell line employed a polymer-based precipitation (PBP) technique for EV isolation, which resulted in a reduction in operation time. Although some non-specific molecules and cell debris may contaminate the exosome pool, the concentrations of these contaminants are lower compared to other isolation methods, such as ultracentrifugation and ultrafiltration. Moreover, the yield of EVs harvested using the PBP method was found to be higher than with other methods, surpassed only by ultrafiltration. The PBP technique also offers several advantages, including reduced protein contamination, the ability to isolate EVs using small sample volumes, and the preservation of EV activity [33]. Furthermore, affinity-based methods for EV isolation have also been developed, as they rely on more specific bindings [34].

EVs can serve as biomarkers to reflect disease status, as they typically contain compositions similar to those of the parental cells. There have been numerous reports indicating that various cancer types could secrete distinct subtypes of EVs as part of their pathological processes. The use of EVs, particularly exosomes, in detecting cancer pathology is beneficial, especially for cancer types whose symptoms may only manifest at advanced stages. For instance, ovarian cancer, one of the most harmful cancer types, can be detected using cancer antigen 125 (CA125) as a biomarker [35]. However, this biomarker alone is insufficient for diagnosis due to its low sensitivity and potential complications related to other disease conditions. Exosomes derived from ovarian cancer have gained attention because they contain specific proteins involved in metastasis and oncogenesis pathways [36].

In studies on epithelial ovarian carcinoma, protein levels in patient-derived exosomes were found to be increased, correlating to those measured in ovarian cancer tissues. This protein profile was further analyzed and suggested as a potential cancer biomarker [37]. Additionally, microRNA profiles of ovarian cancer-derived microvesicles have been reported to correlate with cancer stages, thus making them potentially non-invasive diagnostic tools [38]. This concept of utilizing genomic material-containing EVs can be used to improve the survival rates of patients with other cancers of poor prognosis, including pancreatic cancer [39].

Many researchers have increasingly focused on the proteomic analysis of EVs, which is a fundamental approach to biomarker study. Comparing cancerous groups to control (non-cancerous) groups helps to identify disease status by distinguishing proteomic differences. Studies in colorectal cancer, gastric cancer, ovarian cancer, and melanoma have revealed that changes in protein levels are significantly associated with cancer pathogenesis, including metastasis, invasion, growth, and angiogenesis [38,40,41,42]. The fact that these EVs can stimulate cancer formation in other organs highlights their roles in metastasis. In addition, EV production and secretion in cancer cells differ significantly from those of normal cells. These changes have been reported as a result of the tumor microenvironment’s (TME) hypoxic conditions. The exosome secretion relates to cellular processes that promote tumors. Their roles mainly involve transporting harmful materials from tumor sites, spreading them to healthy tissues, and disrupting the immune balance [43]. The changes in size of EVs are also detectable across various cancers. Studies on breast cancer have demonstrated that small EVs harvested from the plasma portion of breast cancer patients are notably smaller in size compared to those from healthy individuals [44,45]. This size reduction has also been observed in the EV pool isolated from patients with pancreatic ductal adenocarcinoma [46]. Conversely, an increase in the size range of microvesicles has been observed in gastric cancer patients, with an elevated amount during advanced stages [41].

The tumor protein p53 (TP53) gene, one of the most well-known regulators associated with carcinogenesis, has been reported as a key factor influencing EVs. A study on colorectal cancer (CRC) demonstrated the relationship between TP53 and exosome formation. Smaller exosomes were detected in TP53-knockout and TP53-mutated CRC cells compared to those from TP53-wild type cells. Proteomic analysis of the exosomes derived from TP53-disrupted cells revealed downregulation in the expression of hepatocyte growth factor-regulated tyrosine kinase substrate (HGS). This finding was correlated with functional studies, confirming that the reduced expression of HGS was responsible for the size reduction of exosomes. Therefore, the size of exosomes from CRC was also decreased, as observed in breast cancer [47].

One of the major advantages of using EVs as biomarkers is their capacity to assess multiple pathological abnormalities from a single biological specimen. This capacity is particularly useful for diagnostic purposes when diseases originate from a closely related organ system. Blood, particularly the plasma and serum fractions, serves as the primary source for biomarker testing, highlighting the potential of a single-source specimen in disease diagnostics [48]. For example, an investigation of diagnostic method in prostate cancer showed that prostasomes, the microvesicles released from prostate cells, can be used as a biomarker with a less invasive approach as they can be harvested from plasma. The comparison between the prostasomes from cancer patients and healthy individuals revealed that a higher level of prostasomes was detected in cancer patients [49]. In addition to the universal blood specimen, other samples, such as urine, cerebrospinal fluid (CSF), and saliva are alternatively used for biomarker assessment. With advancements in investigative techniques, even more specialized specimens like bronchoalveolar lavage fluid, semen, and peritoneal fluid have been included [48].

Urine, one of the most common specimens due to its non-invasive collection process, can be used to investigate urine-derived EVs for various cancerous pathologies, including those in organs such as bladder and prostate cancer [50,51,52]. The detection of urinary EVs directly provides insights into nephron status, offering valuable information on kidney diseases [53]. Besides the urinary system, previous research has demonstrated the potential of using urine to detect diseases originating from more distant organs. In addition to EVs harvested from traditional sources like CSF and blood, a proteomic study of urine-derived EVs has revealed their potential as a non-invasive source of biomarkers reflecting the neurological status of Parkinson’s disease (PD) [54,55]. The diagnostic application of exosomes is promisingly beneficial not only for diagnosis but also for the development of therapeutic strategies.

### 3.2. Extracellular Vesicles for Tracking Cancer Progression

EVs are recognized for their role in intercellular communication and disease progression. Predicting disease progression is inevitably as important as diagnosing diseases. The application of EVs also holds significant promise in this regard, helping clinicians in diagnosing and evaluating disease status.

EVs are also manipulated to become a mediator for disease progression, being integral components of disease development. Primarily, cancer progression can be identified using the stage-specific proteins carried by EVs. For example, previous analyses of exosomes isolated from urine have revealed insights into the progression of bladder cancer. Researchers found a correlation between an increased level of the specific protein Epidermal Growth Factor-like repeats and Discoidin I-Like Domains 3 (EDIL-3) and cancer angiogenesis and migration, mediated via epidermal growth factor receptor (EGFR) activation [50]. In addition, exosomes containing EDIL-3 play a significant role in the metastasis process of breast cancer, affecting nearby organs such as the lungs. The discovery of high EDIL-3 concentration in metastatic breast cancer suggests that this protein could potentially serve as a stage-specific biomarker, detectable through circulating EVs [56]. Furthermore, different stages of breast cancer were relevant with variations in the number of EVs and the plasma levels of the signaling component, such as focal adhesion kinase (FAK) and EGFR [45].

Elevated amounts of EVs have been reported in various diseases including different cancers. Previous research on plasma-derived microvesicles in gastric cancer patients demonstrated higher quantities across all cancer stages, with even higher levels observed in more advanced stages compared to those in healthy individuals. Additionally, increased surface charges and the presence of tumor markers were notably detected in gastric cancer patients [41]. The changes in EV level also reflect the different stages in prostate cancer. Prostasomes can be used to distinguish cancer severity, separating the low-grade cancer from the higher-grade cancers [49]. Promisingly, analyzing EVs to monitor disease progression provides numerous advantages, including prognosis assessment, the development of treatment strategies, and the optimization of drug development protocols.

### 3.3. Extracellular Vesicles for Monitoring Treatment Response

In addition to their use in cancer prognosis and diagnosis, the clinical significance of EVs extends to monitoring treatment responses and evaluating treatment management. After applying the treatment, it is essential to consider not only the response but also the potential resistance development. Assessing responses to specific treatments ensures the efficacy of the therapy and informs future treatment planning. Normally, various clinical tools, including computed tomography (CT) scans, magnetic resonance imaging (MRI), and tissue biopsy, are used for tracking treatment effectiveness. However, these methods are invasive, and the treated patients often exhibit reduced sensitivity to the CT scans. Consequently, the development of alternatives with less invasive approaches for treatment response detection is essential [57].

Making use of EVs’ unique characteristics, which function as natural intercellular messengers, may provide a significant advantage. Previous studies on the metastasis of breast cancer have highlighted the potential real-time monitoring approaches used to forecast treatment outcomes and support personalized treatment planning [57,58]. In the studies on breast cancer, carcinoma antigen 15-3 (CA15-3) was useful for tracking treatment response and detecting cancer recurrence. This is particularly beneficial where radiological monitoring is not practical. However, this concept of using EVs as a biomarker has limitations, especially when critical treatment decisions need to be made [59,60]. Therefore, there is still room to further develop EV-based detection methods.

The protein contents of EVs have been extensively studied for predicting treatment response. For example, in the clinical management of head and neck squamous cell carcinoma (HNSCC), the levels of transforming growth factor-beta 3 (TGF-β3) protein were monitored as a biomarker for patient response to chemoradiation therapy (CRT). Higher levels of TGF-β3 were detected in non-responsive patients, compared to those levels in CRT-responsive patients [61]. Protein profile in circulating EVs is also valuable for the management of breast cancer treatment, as the method was developed to monitor and predict the treatment response. Eight significant proteins were identified as potential biomarkers and can be used to distinguish the metastatic stage from the non-metastatic stage, with an accuracy percentage of almost 90% [57]. Using EVs in treatment response tracking is also beneficial, as only liquid biopsy is required. The circulating cellular components include tumor cells, genetic materials, and EVs [62]. Genome-wide association studies (GWAS) have also been used for biomarker analysis. Various genes were investigated and evaluated to assess response, as well as toxicity to the treatment of urinary bladder cancer (UBC) [63].

Furthermore, another application of EVs is the prediction of chemoresistance. EVs isolated from the serum of human epidermal growth factor receptor 2 (HER2) breast cancer patients exhibit a higher level of HER2. The resistance is the result of the binding between trastuzumab, a cancer drug, and HER2, eventually leading to changes in protein expression within EVs. Therefore, the analysis of protein profiles from EVs can be valuable for predicting chemoresistance [62].

## 4. Cancer Immunotherapy and Extracellular Vesicles

### 4.1. Immune Mechanisms

#### 4.1.1. Anti-Tumor Immunity

**Figure 3:**
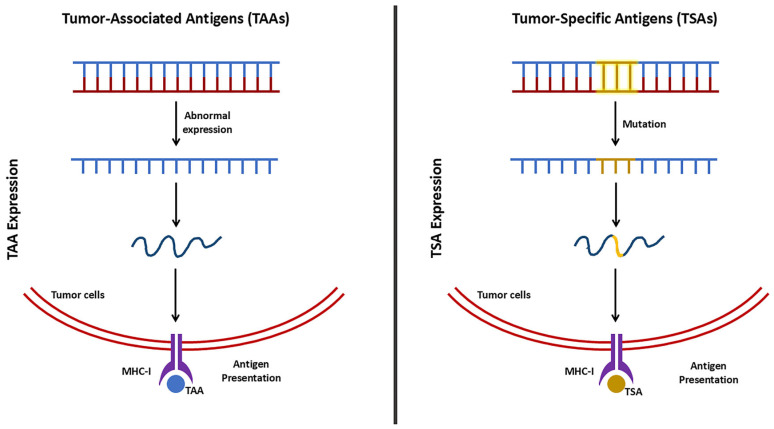
Differences between tumor-associated antigens (TAAs) and tumor-specific antigens (TSAs). Abbreviations: TAAs: tumor-associated antigens; TSAs: tumor-specific antigens; MHC-I: major histocompatibility complex class I.

Tumor cells contain specific antigens, which can be classified into two types, tumor-associated antigens (TAAs) and tumor-specific antigens (TSAs) (Figure 3) [64]. TTAs are normal proteins with overexpressed levels, compared to the levels in normal cells, whereas TSAs occur due to mutations corresponding to the tumor cells. Therefore, TTAs are of particular interest because they are not limited to one specific tumor type [65]. In anti-tumor immunity, TAAs undergo various processes and are presented on the surface of tumor cells via loading onto major histocompatibility complex (MHC) class I and class II molecules. The antigen-MHC I complex is directly recognized by T cell receptors (TCRs) of cytotoxic T cells (CD8^+^ T cells), leading to the direct killing of tumor cells. On the other hand, the antigen-MHC class II complex is recognized by CD4^+^ helper T cells (Th cells) with the assistance of antigen-presenting cells (APCs), which further stimulates additional immune responses, including the activation of CD8^+^ T cells and B cells [66].

In addition to directly activating T cells, various immune cells respond to the presence of tumor antigens. Classic antigen-presenting cells, such as B cells, dendritic cells (DCs), and macrophages, also interact with antigens in a similar manner. B cells recognize TAAs by the B Cell Receptor (BCR) and can either act as antigen-presenting cells, presenting TAAs via MHC molecules to T cells (T cell-dependent immunity), or develop into memory B cells (T cell-independent mechanism). In the latter case, CD27 on memory B cells interacts with CD70 on CD8^+^ T cells, leading to the induction of an antigen-independent T cell response [67,68]. DCs are important immune cells that bridge innate and adaptive immunity [69]. After capturing TAAs, DCs become activated, process the antigens, and present them to T cells using MHC molecules [70]. Similarly, macrophages phagocytose, process, and present TAAs to T cells [71].

#### 4.1.2. Immunosuppressive Mechanisms in Tumor Microenvironment

Inflammation caused by the presence of tumors leads to the accumulation of various immune cells. Despite the ability of the immune system to detect and eliminate tumors, tumors themselves have developed escape mechanisms, one of the hallmarks of cancer progression that allow them to evade immune detection and destruction [3]. Tumors employ a range of immunosuppressive strategies to counteract the anti-tumor immune response.

TME refers to the components surrounding cancerous tumors, consisting of various tumor-derived metabolites and infiltrating immune cells, with fibroblasts as a major component [72]. TME is not only the surroundings of the tumor but also possesses immunosuppressive properties that support the key processes in tumorigenesis, including growth, metastasis, and invasion [73]. Additionally, TME contributes to drug-resistant mechanisms and their ability to escape anti-tumor immunity [74]. Tumors recruit various immunosuppressive cells into the TME as a key strategy to escape the immune system. These immunosuppressive cells mainly include myeloid-derived suppressor cells (MDSCs), tumor-associated macrophages (TAMs), and regulatory CD4^+^ T cells (Tregs). Their main targets predominantly involve several immune cells, including CD8^+^ T cells and natural killer (NK) cells [75].

MDSCs are a heterogeneous population of cells primarily responsible for immunosuppression. Due to their induction and influence within the TME, they exhibit inhibitory activities against T cells, B cells, and NK cells [3,75]. MDSCs are classified into two subtypes based on their origins, morphologies, and phenotypes: monocytic MDSCs (M-MDSCs) and polymorphonuclear MDSCs (PMN-MDSCs). Although both MDSC subtypes share some overlapping features, they have their unique characteristics [75,76]. Research indicates that M-MDSCs exhibit stronger immunosuppressive properties compared to PMN-MDSCs [77]. Both M-MDSCs and PMN-MDSCs interfere with immune regulation through various pathways. These include the upregulation of the signal transducer and the activation of transcription (STAT) proteins, such as STAT1, STAT3, and STAT6, as well as the endoplasmic reticulum (ER) stress pathway, nuclear factor kappa B (NF-κB), and lipid oxidation. They also downregulate cyclic adenosine monophosphate (cAMP), interferon (IFN)-8, prostaglandin E synthase (PTGES), cyclooxygenase-2 (COX-2), and CCAAT-enhancer-binding protein-β (C/EBPβ) [75]. However, the activity of MDSCs is influenced by various tumor factors, including the types and stages of tumors [3].

The primary mechanism by which MDSCs inhibit T cell activation occurs through two pathways: arginase 1 (ARG1) and inducible nitric oxide synthase (iNOS). The upregulation of ARG1 in MDSCs causes a depletion of L-arginine, an essential metabolite for T cells, which further leads to the disruption of the CD3-*ζ* chain of T cells, ultimately resulting in impaired T cell function, blocked proliferation, and reduced cytokine production [78]. This phenomenon is supported by elevated levels of ARG1 observed in various cancer patients, including those with colon, prostate, lung, and breast cancers [79]. Additionally, increased iNOS levels are another distinctive feature of MDSCs, with higher iNOS expression found in M-MDSCs [80]. The resulting increase in nitric oxide production can nitrate TCRs, hindering T cell activation and proliferation, ultimately reducing the antitumor response [81]. MDSCs also generate elevated levels of reactive oxygen species (ROS) which self-stimulate the vascular endothelial growth factor (VEGF) in MDSCs, further recruiting more MDSCs into the TME [82]. ROS play a role in the expansion of MDSCs and affect T cells by altering their redox state, inhibiting TCR signaling, and inducing T cell apoptosis [75,83].

The secretion of cytokines, TGF-β and interleukin (IL)-10, by MDSCs represents another mechanism in which MDSCs exert their immunosuppressive effects. These cytokines suppress CD8^+^ T cell activation while simultaneously promoting and recruiting Tregs, leading to a reduction in antitumor immunity [84,85]. NK cells are also suppressed by TGF-β, which is stimulated by prostaglandin E2 (PGE2) [86]. Additionally, MDSCs contribute to TME modulation by altering the expression of MHC class II molecules on DCs and macrophages, which interferes with antigen presentation to CD4^+^ T cells, further weakening the immune response [87,88]. MDSCs play a critical role in establishing an immunosuppressive TME, and they utilize multiple mechanisms to inhibit anti-tumor immunity and ultimately promote tumor growth and metastasis.

TAMs are another type of immunosuppressive cells that are recruited and modulated within the TME. TAMs are found in a greater amount than other immune cells. TAMs can be categorized into two subtypes: M1 (pro-inflammatory) and M2 (anti-inflammatory) [89]. M2 TAMs play a key role in immunosuppression within the TME through various mechanisms, including the secretion of cytokines such as IL-6, IL-10, and TGF-β, which promote the recruitment of Treg to the site. Additionally, TAMs express programmed cell death-ligand 1 (PD-L1), which inhibits the function of CD8^+^ T cells [90,91]. Furthermore, TAMs contribute to tumor progression by promoting angiogenesis and metastasis through the secretion of VEGF. The chemokine (C-C motif) ligand 18 (CCL18), also secreted by TAMs, facilitates tumor migration alongside TGF-β. A distinctive characteristic of the tumor extracellular matrix (ECM) is its density, which limits the effectiveness of external treatments and increases tumor aggressiveness. TAMs regulate ECM remodeling by secreting proteases that degrade collagen fibers and enzymes that enhance ECM stiffness, promoting tumor progression [90]. Furthermore, TAM-secreted TGF-β has been associated with the impaired function of NK cells [92].

As a part of immune homeostasis, Tregs function as regulators for self-antigen immunity by employing various mechanisms to maintain immune balance. Tregs secrete several immunosuppressive cytokines, including IL-10, IL-35, and TGF-β, which inhibit the activity of effector T cells (Teffs) [93]. Additionally, Tregs express the checkpoint protein cytotoxic T-lymphocyte-associated protein 4 (CTLA-4) at high levels, which downregulates CD80 and CD86 molecules on APCs, thereby limiting T-cell-mediated immunity [94]. Tregs also constitutively express CD25, the IL-2 receptor subunit-α, depleting IL-2, and thus reducing the availability of IL-2 to activate T cells, B cells, APCs, and NK cells [95]. Furthermore, Tregs produce adenosine, a small molecule that suppresses the immunological functions of Teff cells and APCs [93].

#### 4.1.3. Immune Checkpoint Pathways in Tumor Immune Evasion

Tumors also upregulate immune checkpoint molecules as a defensive mechanism against the immune system. Under normal conditions, immune checkpoints play a key role in self-regulation by preventing excessive immune responses, autoimmunity, and cell damage caused by immune activities. However, tumors hijack these pathways to suppress immune activity. Various immune checkpoint molecules are involved in immune checkpoint cascades, with the most well-known immune checkpoints being programmed cell death protein 1 (PD-1), PD-L1, and CTLA-4 [96].

The PD1/PD-L1 signaling pathway is one of the most prominent immune checkpoints. In tumor immune evasion, PD-L1 (or CD274) is expressed on the surface of various cells, including macrophages, APCs, and DCs and is found at elevated levels on tumor cells as part of their immune tolerance mechanism [97]. Conversely, PD-1 (or CD279) is expressed on B cells and T cells, particularly activated T cells [98]. When T cells recognize tumor antigens, they upregulate PD-1 and secrete interferon-gamma (IFN-γ). Tumor cells exploit this response by upregulating PD-L1, which is further induced by secreted IFN-γ within the TME. Upon PD1/PD-L1 interaction, tyrosine residues of PD-1 undergo phosphorylation, which consequently disrupts downstream signaling pathways, including phosphatidylinositol 3-kinase (PI3K) and rat sarcoma virus (RAS), thereby inhibiting the production of cytokines critically essential for T cell activation [97]. This interaction predominantly suppresses cytotoxic T lymphocyte (CTL) activity, leading to reduced CTL activation and proliferation, as well as diminished secretion of tumor-toxic cytokines, ultimately resulting in tumor immune resistance [99]. Further studies have revealed that PD-1 is upregulated in tumor-specific T cells, further impairing the anti-tumor immune response [98].

CTLA-4 is an important immune checkpoint molecule, primarily found on T cells. It shares a similar mechanism with PD-1 in modulating T cell activation. They both are responsible for preventing the overactivation of the immune response. Their mechanisms involve the inhibition of serine/threonine protein kinase (Akt) activity, a key element in glucose metabolism during T cell activation [100]. For the full activation of T cells, the binding of TCR to antigens presented by MHC requires a costimulatory signal, which occurs through the interaction between CD28 on T cells and B7 molecules (B7-1 (CD80) and B7-2 (CD86)) on activated APCs [101]. However, the immune system has its own checkpoint mechanisms used to regulate and prevent overactivation. In T cells, this regulation occurs via two main pathways: through the CD28 signal or the induction of CTLA-4 [101]. With constitutive expression on Tregs, CTLA-4 competes with CD28 for binding to B7 molecules on APCs with higher affinity, thereby blocking T cell co-stimulation [102]. Tumors exploit this pathway, along with the presence of Tregs, to evade immune detection. Tregs are found in higher amounts in tumors compared to healthy tissues [103]. In TME, the upregulation of CTLA-4 on Tregs reduces the availability of B7 molecules for CD28 binding, which is necessary for proper T cell activation. This reduction in co-stimulatory signaling limits T cell activation and proliferation, weakening anti-tumor immunity and allowing tumor growth to proceed unchecked [104]. Additionally, prolonged exposure to tumor antigens within TME can lead to T cell exhaustion. Exhausted T cells show increased expression of inhibitory receptors, including CTLA-4 and PD-1, and reduced production of effector cytokines, such as IL-2, tumor necrosis factor (TNF-α), and IFN-γ, further weakening the immune response against the tumor [105]. Furthermore, CTLA-4 has been found to be expressed on various tumor cells [106]. Studies have demonstrated a correlation between CTLA-4 expression and the survival rate of cancer patients, including those with non-small cell lung cancer (NSCLC), suggesting its potential as a prognostic marker in cancer treatment [107].

### 4.2. Extracellular Vesicles in Cancer Immunotherapy

EVs have been developed for use in drug delivery systems due to their potent biological carrier properties. Similar to other drug development objectives, EVs aim to enhance the pharmaceutical properties of drugs, including improving pharmacokinetics and pharmacodynamics, increasing drug efficacy, and minimizing potential toxicity and side effects. Additional advantages are improved drug solubility and a cell-free approach, which makes them easily reproducible and scalable [108].

EVs are natural carriers for various biomolecules. In addition to transporting therapeutic biomolecules such as DNAs, RNAs, proteins, and small molecules, they are also valuable for targeted therapies. Active drugs can be loaded into EVs to increase bioavailability and reduce unspecific side effects. Methods for loading drugs into EVs can be categorized into two approaches: endogenous and exogenous. The endogenous approach involves the manipulation of EV-producing cells, including changes through genetic engineering, to produce EVs with desired components. However, the modifications for the endogenous technique are somehow limited for those involved in the biological contents [108].

For drug encapsulation apart from using nucleic acids and proteins, various exogenous techniques have been developed. In this method, EVs are first produced and harvested from biological sources, and the desired contents are then incorporated using either chemical or mechanical methods. The loading techniques are further divided into passive and active methods. Passive loading involves incubating the desired drug with either EVs (direct) or EV-producing cells (indirect). On the other hand, active loading requires external forces, such as sonication, extrusion, electroporation, or temperature changes. Some membrane permeabilizers, like saponin, are also used to create pores in the membrane, enhancing drug loading capacity [109]. The surface components of EVs are important for their biological functions and can be modified depending on therapeutic purposes. According to their biogenesis, EVs carry surface markers from their original cells, which can influence their effects on targeted sites, either positively or negatively. Apart from EV sources, administration route plays a significant role in the biodistribution of EV-based drugs [16,110]. Therefore, choosing the appropriate EV source and route of administration is crucial for therapeutic efficacy.

#### 4.2.1. Extracellular Vesicles for Targeted Delivery

EVs significantly impact the development of various biomedical applications, as both native and engineered EVs can be applied for therapeutic purposes. Carrying protein and components from the parental cells gives the distinct properties for naturally derived EVs for various therapeutic applications, with some adjustments [35]. Some naturally derived EVs, such as those secreted from tumor cells, DCs, and mesenchymal stem cells, have intrinsic immunostimulatory properties that can enhance immune responses [111,112,113].

Additional modifications to EVs can enhance targeting and specificity, leading to increased therapeutic efficacy. EVs can be optimized for specific applications through the use of genetic engineering. Furthermore, strategies on the enhancement of T cell function offer promising immunostimulatory outcomes. IL-2 has been extensively studied as an immune effector that promotes the proliferation and activation of various immune cells, including CD8^+^ T cells, CD4^+^ T cells, and NK cells [95,114]. Therefore, IL-2 has drawn attention as one of the most potent immunotherapeutic agents, with its ability to stimulate and enhance immune responses. Notably, IL-2 was the first interleukin approved by the U.S. FDA as a cancer immunotherapeutic agent for metastatic renal cell carcinoma and metastatic melanoma [115].

The important roles of IL-12 involve maintaining the homeostasis of the immune response through the induction of differentiation and proliferation of both Teffs and Tregs. Tregs can suppress Teffs, leading to the induction of the overall anti-tumor activity. Tregs are particularly sensitive to IL-2 at a very low concentration due to their constitutive expression of CD25 (IL-2 receptor subunit-α). Upon IL-2 binding, CD25 undergoes conformational changes that help maintain a balance between Teffs and Tregs. These two cell types have opposing immunological properties, which are essential for immune system regulation. Therefore, IL-2 possesses a dual effect, acting on both Tregs and Teffs in a dose-dependent manner, and has been explored as a potential immunotherapy tool for autoimmune diseases [116].

Many strategies have been developed to enhance the therapeutic benefits of IL-2, by focusing on increasing its effects on Teffs and NK cells while minimizing its impacts on Tregs. Combination therapies have attracted attention as a means to overcome these limitations. In one particular approach, IL-2 was attached to the surface of EVs, leading to anti-cancer effects via the induction of CD8^+^ T cell cytotoxicity and subsequent tumor inhibition, without causing the effect on Tregs. Additionally, microRNA (miRNA) profiles were modified to reduce PD-L1 expression, further enhancing the anti-cancer properties [117].

#### 4.2.2. Extracellular Vesicles as Vaccines

Vaccines used for cancer treatment primarily based on the presentation of tumor antigens to APCs subsequentially induce the immune response that can counteract cancers [118]. Various tumor components are applied and incorporated into the vaccine to activate the immune system, including the tumor membrane and even whole tumor cells as the sources of tumor antigens [119]. EVs derived from tumor cells and APCs are increasingly used as vaccines in various tumor immunotherapy applications. Due to their low immunogenicity, EV-based nanovaccines offer several advantages over other delivery systems. Compared to commonly used delivery platforms, such as lipid nanoparticles (LNP), EV-based vaccines exhibit enhanced delivery efficacy and long-term stability within the circulation. LNPs, while effective for many applications, face limitations in delivering certain therapeutic proteins, particularly transmembrane proteins, which are crucial for immune recognition but cannot be presented in their natural form by LNPs. This limitation has notably brought significant attention to EVs as a promising delivery tool for these proteins [120].

**Figure 4:**
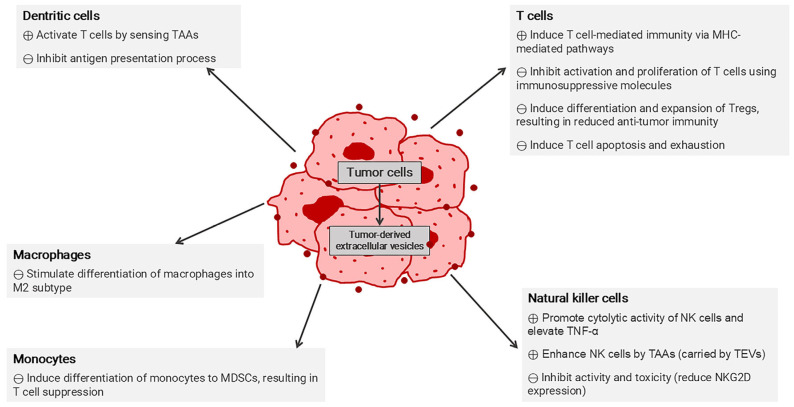
General immunological effects of tumor-derived extracellular vesicles. Tumor-derived extracellular vesicles can exert both positive (⊕) and negative (⊖) effects on immune cells. These effects may result in either immunostimulatory or immunosuppressive outcomes, and the mechanisms involved can be either direct or indirect. Abbreviations: TEVs: tumor-derived extracellular vesicles; TAAs: tumor-associated antigens; MHC: major histocompatibility complex; Tregs: regulatory T cells; MDSCs: myeloid-derived suppressor cells; NK cells: natural killer cells; NKG2D: natural killer group 2 member D; TNF-α: tumor necrosis factor alpha.

Within the TME, tumor-derived EVs act as intercellular communicators, influencing tumor pathology by promoting tumor cell survival, growth, and invasion via the stimulation of T cells. Overall, in terms of immune response, tumor-derived EVs affect various immune cells, contributing to both immunostimulatory and immunosuppressive properties. Different effects of tumor-derived EVs via direct and indirect pathways are shown in Figure 4. Despite their role in tumor progression, EVs also hold significant potential for immunotherapy. These tumor-derived EVs play a role in the antigen presentation process through MHC molecules, thereby supporting T cell activation and maturation and eventually promoting anti-tumor immune responses [111,121,122].

Various strategies, such as surface modifications, enhance their specificity and delivery efficacy. For instance, in the previous study, glycan-conjugated melanoma-derived EVs were used to improve targeting, delivering TAAs to DCs and inducing both CD4^+^ and CD8^+^ responses [123]. Another surface modification involves the use of CARs to improve the binding specificity. Genetic modification was performed using exosomes from B16 melanoma cells with the TAAs and bacterial antigens, resulting in increased immunostimulation and tumor growth inhibition [124].

DCs particularly serve as a primary source of therapeutic EVs due to their antigen-presenting and immune-activating properties. DC-derived EVs naturally display immune molecules, such as MHC and other stimulatory molecules, that play essential roles in activating CD4^+^ T cells, CD8^+^ T cells, B cells, and NK cells [125]. One study demonstrated the intrinsic anti-tumor properties of DC-derived EVs, notably through NK cell activation, as observed in NSCLC patients [126]. Genetic modifications to DCs can lead to the release of EVs containing CTLA-4, thereby leading to the induction of T cell-mediated immune responses [127]. Consequently, DC-derived EVs have been extensively studied and are currently featured in multiple ongoing clinical trials.

#### 4.2.3. Extracellular Vesicles for Overcoming Immunosuppressive Tumor Microenvironment

TME is a complex system in which molecular components are adapted to contribute to tumorigenesis. A similar phenomenon occurs with EVs derived from tumor and immune cells, which express immune checkpoint proteins associated with tumor-promoting mechanisms [128]. The secretion of EVs is notably higher under hypoxic conditions in the TME, further facilitating tumor progression [43].

To manage the immune balance, the immune system normally regulates itself by the surveillance system known as the immune checkpoint mechanism, using checkpoint proteins to control and prevent excessive or self-directed immune responses. However, tumors can utilize these mechanisms by producing proteins that mimic those of normal cells, allowing them to evade immune detection. Therefore, researchers have developed strategies to block these interactions between tumor-derived proteins and immune checkpoint proteins. ICIs are drugs designed to inhibit the binding of checkpoint proteins, thereby enabling immune cells to recognize and attack tumor cells, eventually leading to their elimination. The primary function of ICIs is to restore the immune system’s ability to detect, target, and eliminate tumor cells by disrupting checkpoint protein interactions [96].

Although ICIs have been developed to stimulate the body’s immunity against tumors, resistance to ICI therapies has emerged. This is one reason why ICIs are not universally effective, with some emergence of tolerance. Tumors have their own mechanisms to control the ICI treatment. Various EVs contain immune checkpoint proteins, such as those expressing PD-L1. PD-L1 is normally found on a range of immune cells, as well as tumor cells [97]. Therefore, EVs released from tumor cells carry over this immune checkpoint protein, as shown in studies on various cancers, including NSCLC and breast cancer. PD-L1 molecules on EVs can suppress the immune response via the PD-1/PD-L1 pathway. Binding to PD-1 on T cells leads to several inhibitory pathways, similar to other inhibitory mechanisms involving CD28 and NF-κB signaling, eventually inhibiting the proliferation of T cells [129,130]. EVs containing PD-L1 potentially cause the induction of an immunosuppressive cytokine, IL-10 [131]. Moreover, the expression level of PD-L1 on EVs in NSCLC correlates with levels in the tumor itself, suggesting that the origin of these EVs is tumor cells [129]. Further investigation has demonstrated that PD-L1 can be transferred to breast cancer cells lacking PD-1, leading to enhanced immune suppression [130].

**Figure 5:**
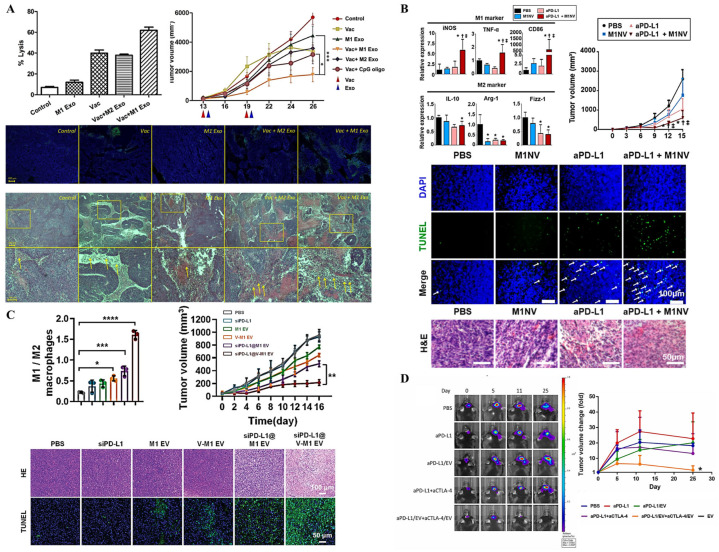
Extracellular vesicle efficacy in tumor inhibition. (**A**) Extracellular vesicles derived from the M1 subtype of macrophages exhibit immune induction ability and enhance tyrosinase-related protein 2 (TRP2) vaccine efficacy against B16F10 melanoma: lysis percentage reflecting the killing efficacy (Student’s *t* test), tumor volume observed in mm^3^ (*n* = 6–8, one-way ANOVA, * *p* < 0.05, *** *p* < 0.001), representative TUNEL assay images with apoptotic cells (labelled in green) and total cells (labelled in blue) (scale bar, 200 µm), and representative H&E staining images of tumors at the endpoint (scale bar, 200 µm (upper panel); scale bar, 100 µm (lower panel, the magnified images of the rectangles in the upper panels); arrows define the infiltrating immune cells. All data are presented as mean ± SD. Reproduced with permission [132]. Copyright 2017, Elsevier. (**B**) Nanovesicles derived from the M1 subtype of macrophages (M1NV) can polarize M2 to M1 subtype and enhance the anti PD-L1 (aPD-L1) therapeutic efficacy: the relative expression of M1 marker (iNOS, TNF-α, CD86) and M2 marker (IL-10, Arg-1, Fizz-1) mRNA (*n* = 3, one-way ANOVA and Tukey’s significant difference post hoc test), tumor volume measured in tumor mice (*n* = 5–13), fluorescent images of the representative areas using TUNEL assay demonstrating apoptotic cells (labelled in green) and total cells (labelled in blue) measured in tumors 24 h after the end of the treatment (scale bar, 100 µm), and H&E staining images of tumor tissues (scale bar, 50 µm). All statistical significance was * *p* < 0.05 compared with PBS, † *p* < 0.05 compared with M1NV, ‡ *p* < 0.05 compared with aPD-L1. All data are presented as mean ± SD. Reproduced with permission [133]. Copyright 2018, American Chemical Society. (**C**) Extracellular vesicles from the M1 subtype of macrophages combined with pH-sensitive glycoprotein and anti-PD-L1 siRNA, demonstrating the efficacy to polarize M1 to the M2 subtype and reduce tumor growth in vivo: M1/M2 macrophage ratio, tumor volumes (measured in mm^3^), and H&E and TUNEL assay. All data are presented as mean ± SD. All statistical significance was * *p* < 0.05, ** *p* < 0.01, *** *p* < 0.001, and **** *p* < 0.0001 (*n* = 3). Reproduced with permission [134]. Copyright, 2020, Wiley. (**D**) Combination of extracellular vesicles from calcium phosphate particle-induced macrophages with ICI antibodies showing the enhanced anti-tumor efficacies in glioma mice: representative images of tumors from day 0 to day 25 and changes in tumor volume (*n* = 8–18, Bonferroni post hoc test). * refers to a significant difference (*p* < 0.05), compared to PBS and aPD-L1 groups. All data are presented as mean ± SD. Reproduced with permission [135]. Copyright, 2024, Elsevier.

However, EVs remain useful and possess unique properties for modulating TME. According to the characterization macrophages with TME, they are divided into two subtypes, M1 and M2 [89]. Previous studies have demonstrated that EVs derived from M1 macrophages can induce proinflammatory response and inhibit tumor growth. Upon subcutaneous administration, M1-derived EVs are taken up by DCs and macrophages, leading to the release of proinflammatory cytokines, including IL-6 and IL-12, which promote the T-helper cell type 1 (Th1) response. Furthermore, when combined with the use of the tyrosinase-related protein 2 (TRP2) tumor vaccine, M1-derived EVs can enhance the vaccine’s anti-tumor efficacy, as shown by higher killing capability, reduction in tumor size, the amount of apoptotic cells, and the histological images of tumors (Figure 5A) [132]. Studies conducted both in vitro and in vivo using nanovesicles derived from M1 have shown that these vesicles can successfully repolarize M2 macrophages into the M1 subtype. Additionally, when combined with ICI treatment, such as anti PD-L1, M1-derived nanovesicles further enhanced anti-tumor efficiency, as evidenced by a greater reduction in tumor size and elevated apoptosis compared to the treatment with either M1 nanovesicles or anti PD-L1 alone (Figure 5B) [133].

In TME, tumor-derived EVs play a prominent role in establishing an immunosuppressive environment. In addition to carrying PD-L1 [129,130], tumor-derived EVs induce various cellular components involved in tumor progression, including the conversion of immature myeloid cells (IMC) into MDSCs, which contribute to immunosuppression, by disrupting T cell functions [79,136].

Tumor-derived EVs affect a wide range of immune cells, not just a few specific types. Various cancers have developed the secreted EVs that promote the polarization of macrophages to the M2 subtype, which supports tumor growth. Studies on the cargo being carried by EVs secreted from different tumors highlight their role in driving M2 macrophage polarization. For example, EVs containing miRNA have been shown to induce macrophage polarization, as seen in breast cancers (via miR-138-5p) and lung adenocarcinoma (via miR-3153) [137,138].

Tumor-derived EVs also express CD47, a protein found on tumor cells that normally binds to signal regulatory protein alpha (SIRPα) on macrophages to inhibit phagocytosis. EV-associated CD47 exhibits similar anti-phagocytosis properties, leading to the prevention of EV clearance by monocytes [139]. However, tumor-derived EVs are also recognized as having an important role in immune induction, as they carry TAAs that can promote a T cell-dependent response through antigen presentation on MHC class I molecules of DCs [140]. This has led to efforts to combine tumor-derived EVs with other currently used chemotherapeutic agents to enhance therapeutic efficacy and target specificity [141].

NK cells, which are crucial for tumor-killing capabilities, are also affected by tumor-derived EVs. In hepatocellular carcinoma, circular ubiquitin-like with PHD and ring finger domain 1 RNA (circUHRF1) was observed in the secreted exosomes and involved in inhibiting the secretion of TNF-α and IFN-γ. This subsequently leads to NK cell dysfunction. Additionally, circUHRF1 is involved in resistance to immune checkpoint therapies, such as anti-PD1 [142].

Treg also promotes tumor progression. From the study of EVs derived from HNSCC, PD-L1 on tumor-derived EVs was shown to influence Treg differentiation and function, consequently leading to T cell suppression and immunosuppressive effects [143]. Increased recruitment of Treg into the TME area has been observed in various cancers [144]. Therefore, several strategies have been developed to block Treg activity and recruitment. One key target is CTLA-4, and numerous anti-CTLA-4 drugs have been developed to impede its binding to CD80 and CD86 on APC, thereby allowing T cell response [101]. The approved CTLA-4 blockers for various tumor treatments include ipilimumab (approved in 2011) and tremelimumab (approved in 2022, combined therapy with durvalumab) [145,146].

Another important factor for tumor progression and metastasis is TGF-β, as it associates with various tumor progression processes within TME. As a part of TME modulation, TGF-β plays an important role in immunosuppression, via the secretion from MDSCs and M2 TAMs. TGF-β signaling is a complex pathway that associates with EVs and contributes to the homeostasis of various immune cells [84,85,90,91]. Therefore, there have been attempts to capture and eliminate TGF-β. Some examples of these antagonistic drugs include imatinib mesylate, glitazones, tranilast, and losartan. These anti-TGF-β drugs hinder its production, secretion, and function of TGF-β [147]. EVs have been used as a tool to carry tumor inhibitors. A study on engineered EVs was conducted to modify the outer compartment to carry the TGF-β type II receptor. Together with arginine-glycine-aspartic acid (RGD) peptide, these modified EVs contributed to elevated numbers of CD8^+^ T cells and the ratio of M1/M2 macrophages, eventually resulting in anti-tumor effects and an increased survival rate in glioblastoma mouse models [148]. Another example of EVs on tumor inhibition is the combination with VEGF siRNA and doxorubicin. From the in vitro testing in glioma cells, the reduction of angiogenesis and elevation of tumor apoptosis were observed. The result was more desirable than that of the convention anti-VEGF drug, bevacizumab [149]. Overall, EVs are beneficial for carrying the molecules necessary for tumor inhibition and for modulating TME.

#### 4.2.4. Combining Extracellular Vesicles with Immune Checkpoint Inhibitors

Tumors exploit immune checkpoint mechanisms to evade anti-tumor responses. Therefore, the uses of ICIs to counteract and block these bindings notably allow immune cells to recognize malignant cells, activate immunity, and eventually cause the death of tumor cells [96]. A wide range of ICIs are available on the market, offering potential approaches for tumor immunotherapy. The U.S. FDA-approved available ICI drugs are listed with important clinical trials in Table 2. Among various ICIs, anti-PD1/PD-L1 therapies are the most notable and successful target, having demonstrated efficacy across a wide range of cancer types [99]. Compared to traditional therapies such as chemotherapy, ICIs exhibit higher efficacy due to their direct involvement and association with the immune response [96]. ICIs offer advantages over traditional treatments such as chemotherapy and radiation therapy, as they induce a sustained immune response, leading to longer-lasting effects. However, several limitations have emerged that obstruct the desired therapeutic outcomes, including low response rates, adverse effects, and the emergence of resistance [2,150].

In NSCLC patients, the use of ICIs results in less than a 50% response rate through the mixed lineage kinase domain-like protein (MLKL) and receptor-interacting protein kinase 3 (RIPK3) signaling pathways [167]. Similarly, advanced melanoma treatment targeting CTLA-4 shows a response rate of approximately 40% [151]. Moreover, PD-1/PD-L1 immunotherapy has demonstrated a 23% response rate in advanced colorectal cancer, with notably adverse side effects [168]. Therapeutic outcomes also depend on the presence of specific molecular targets in tumors. For instance, studies of PD-1/PD-L1 immunotherapy have shown that tumors expressing PD-L1 exhibit a higher response rate compared to those without PD-L1 [169].

Although EVs are key contributors to tumor progression, they have been increasingly used in tumor therapies, particularly in combination with immunotherapy, due to several inherent advantages [108]. Efforts have been made to develop novel therapeutic EVs in the combination with ICIs. Below are some applications of immune cell-derived EVs used alongside ICIs.

To induce EV production, various methods have been used to enhance therapeutic potential. For example, studies using LED light therapy have shown that it increases the secretion of DC-derived EVs, which retain the same properties as naturally secreted EVs [113]. Additionally, genetically modified DCs have been used to produce EVs containing anti-CD19 and PD-1, which elevate accumulation in CD19-positive solid tumors and inhibit the PD-1/PD-L1 pathway, ultimately preventing metastasis [170]. Another approach involves combining anti-CTLA-4 with DC-derived EVs, resulting in enhanced T cell responses and the inhibition of tumor-draining lymph node growth [171].

As for the macrophage-derived EVs, EVs secreted from M1 macrophages have been engineered via electroporation to carry PD-L1 siRNA, which result in the inhibition of PD-1/PD-L1 interactions, exhibiting anti-tumor effects (Figure 5C) [134]. In glioma treatment, macrophages were stimulated to produce EVs capable of polarizing macrophages into the proinflammatory subtype. ICIs, including PD-L1 and CTLA-4, were loaded onto macrophage-derived EVs, which enhanced pharmaceutical biodistribution to the brain through nasal administration. This approach demonstrated higher therapeutic efficacy by reducing tumor growth and expansion (Figure 5D) [135].

T cells have been genetically engineered to overexpress PD-1, resulting in the release of EVs carrying PD-1 molecules. These PD-1 EVs bind to PD-L1 on tumor cells, thereby inhibiting the PD-1/PD-L1 pathway and facilitating T cell-mediated anti-tumor immunity. These engineered T cells can also induce tumor cell death through granzyme B (GzmB) and Fas ligand (FasL) pathways [172]. In a study involving melanoma mice, EVs secreted by CD4^+^ T cells were shown to induce potent T cell-mediated immune responses. The anti-tumor effect was further enhanced when IL-2 was used to stimulate CD4^+^ T cells during EV production, resulting in an increased yield of CD4^+^ T cell-derived EVs and further strengthening the CD8^+^ T cell immunity. This study highlighted the innovative potential of manipulating EV production for tumor management [173].

## 5. Conclusions and Future Perspectives

The human body has innate mechanisms that detect and destroy foreign and malignant cells, as well as the regulatory immune system that regulates and monitors immune responses. For tumors to survive, they evolve additional strategies to evade and dysfunction immune detection. Tumors are complex and are composed of heterogeneous cell populations, and they actively manipulate their surrounding environment, known as the tumor microenvironment. These alterations enable immune evasion and facilitate tumor growth and metastasis [73].

Recent advancements have focused on developing novel methods to counteract tumor-induced immunosuppression. ICIs have emerged as a advancement in tumor immunotherapy. The concept of ICIs is that they utilize the immune checkpoint mechanisms by blocking the inhibitory interaction between the tumor-related components, known as immune checkpoint proteins, and the immune cells, thus restoring immune activity against tumors [92]. These therapies have demonstrated long-term efficacy and response. For instance, the monoclonal antibody ipilimumab, which targets CTLA-4 on T cells, became the first FDA-approved ICI in 2011 after a Phase 3 clinical trial showed efficacy in patients with metastatic melanoma [145]. To date, more than ten ICIs are available, targeting pathways such as PD-1, PD-L1, and CTLA-4 across various cancer types.

In addition to ICIs, other approaches for immunotherapy include CAR-T cell therapy, cancer vaccine, and monoclonal antibodies. However, the adverse effects that relate to the use of these therapies need to be monitored and solved. Therefore, there have been various attempts to develop alternative methods to minimize these risks [1]. Like other cells with the body, EVs are also secreted by tumor cells. Tumor-derived EVs are one of many components within TME, in which they lead the immunosuppressive features, contributing to the survival of tumors [72]. In addition to tumor-related biological processes, tumor-derived EVs carry over some molecules from their parental cells, including PD-L1, which counteract PD-1 on T cells, neutralizing the T cell-mediated immune response [129,130,174].

Despite the immunosuppressive properties of tumor-derived EVs, EVs are being explored as therapeutic tools for tumor management. EVs, as naturally derived nanovesicles, have become promising candidates for drug delivery in tumor management. They offer advantages for drug delivery, including biocompatibility, low immunogenicity, and the ability to cross biological membranes. Various types of active components, including proteins, biomolecules, and drugs, can be loaded into EVs to avoid immunogenicity and increase the therapeutic levels. Higher therapeutic doses can also be incorporated into the EVs, leading to higher dosing without causing unpleasant outcomes [108]. Besides using the natural-derived EVs, several techniques, including endogenous or exogenous manipulations, are utilized to for engineering EVs and make them suitable for specific therapeutic applications [108,109].

Combining the current ICIs with other drug delivery approaches, such as using EVs as part of the cell-based approaches, holds potential for improved therapeutic efficacy with some advantages including biocompatibility and more precision in targeting [175]. However, there are significant challenges. In terms of EVs, apart from the complexities of loading active components into EVs, the production and quality control of EVs themselves require careful optimization. EVs are inherently heterogenous; therefore, screening methods are required to filter the undesirable subpopulation of EVs from the whole pool [176]. Standardizing production and purification techniques is critical for scalability. Although EVs have proven to have low immunogenicity, further testing is required to validate this across therapeutic properties. Additionally, further modifications for enhanced EV delivery efficiency with minimized side effects remain an area for future research. A deeper understanding of EV biology could lead to more effective treatments and help to overcome current limitations in immunotherapy.

Overall, increasing comprehension of the mechanisms relating to anti-tumor immune responses is indeed essential. The discovery of new immune pathways could pave the way for novel ICIs and other immune-related approaches, as they are promising methods for inducing overall immune responses to fight against malignant cells. Furthermore, additional major immune checkpoints, such as T cell immunoglobulin and mucin domain 3 (TIM-3) and lymphocyte activation gene 3 (LAG-3), also hold great promise as ICI targets for future anti-tumor therapeutic management and can be approved by the U.S. FDA in the future.

In addition to the development of ICIs, other strategies, including the use of biomaterials, have demonstrated significant improvements in therapeutic outcomes by increasing delivery efficacy and potentially modulating immune-based treatments [177]. Other alternative approaches involve combining multiple methods. This potentially leads to achieve desirable outcomes by targeting various immune pathways. Targeting key immune cells, including Tregs and MDSCs, promisingly induces anti-tumor responses, as these immune cells contribute to the immunotherapy tolerance of tumors. Many research efforts now focus on personalized treatments designed according to each patient’s unique profile. Developing diverse approaches for tumor immunotherapy using the expanded understanding of the complex interactions between tumors and immune cells could be highly beneficial for advancing immunotherapy success.

## Figures and Tables

**Table 1:** Different subtypes of extracellular vesicles and their characteristics.

	Exosomes	Microvesicles	Apoptotic Bodies
**Size**	30–150 nm	40–1000 nm	1–5 µm
**Original source**	Multivesicular bodies and fusion with cell membrane	Outward cleavage of plasma membrane	Protrusion of cell membrane of apoptotic cells
**Specific markers**	CD9, CD81, CD63, TSG101, Alix, HSP70	CD40, integrins, selectins	Phosphatidylserine

**Table 2:** Available immune checkpoint inhibitors for monotherapy approved by the U.S. FDA.

Target	Drug Generic Name (First Approval)	Targeted Cancer (Clinical Trial)	Key Clinical Trial	Reference
**PD-1**	Nivolumab (approved in 2014)	Unresectable/metastatic melanoma	NCT01721746 (CheckMate 037)	[151]
		Advanced/metastatic squamous NSCLC	NCT01642004 (CheckMate 017)	[152]
	Pembrolizumab (approved in 2014)	Advanced melanoma	NCT01295827 (KEYNOTE-001)	[153]
		Advanced NSCLC	NCT01295827 (KEYNOTE-001), NCT01905657 (KEYNOTE-010)	[154,155]
	Cemiplimab (approved in 2018)	Advanced CSCC	NCT02760498	[156]
	Retifanlimab (approved 2023)	Metastatic/locally advanced MCC	NCT03599713 (POD1UM-201)	[157]
	Toripalimab (approved in 2023)	Recurrent/metastatic NPC	NCT02915432 (POLARIS-02), NCT03581786 (JUNIPER-02)	[158,159]
**PD-L1**	Durvalumab (approved in 2017)	Locally advanced NSCLC	NCT02125461 (PACIFIC)	[160]
		Advanced/metastatic urothelial carcinoma	NCT01693562	[161]
	Atezolizumab (approved in 2016)	Urothelial carcinoma	IMvigor 210 (NCT02108652)	[162]
		NSCLC	IMpower110 (NCT02409342)	[163]
	Avelumab (approved in 2017)	Metastatic MCC	JAVELIN Merkel 200 (NCT02155647)	[164]
		Advanced/metastatic urothelial carcinoma	JAVELIN Bladder 100 (NCT02603432)	[165]
	Dostarlimab (approved in 2023)	Endometrial cancer	GARNET (NCT02715284)	[166]
**CTLA-4**	Ipilimumab (approved in 2011)	Malignant/metastatic melanoma	NCT00094653	[145]
	Tremelimumab (approved in 2022, in combination with Durvalumab)	Unresectable HCC	HIMALAYA (NCT03298451)	[146]

Abbreviations: NCT, The National Clinical Trial; NSCLC, non-small cell lung cancer; CSCC, cutaneous squamous cell carcinoma; NPC, nasopharyngeal cancer; MCC, Merkel cell carcinoma; HCC, hepatocellular carcinoma.

## Abbreviations

The following abbreviations are used in this manuscript

CAR	Chimeric Antigen Receptor
FDA	Food and Drug Administration
ICIs	Immune Checkpoint Inhibitors
EVs	Extracellular Vesicles
MISEV	Minimal Information for Studies of Extracellular Vesicles
DNA	Deoxyribonucleic Acid
RNA	Ribonucleic Acid
ILVs	Intraluminal Vesicles
MVEs	Multivesicular Endosomes
CD	Cluster of Differentiation
ELISA	Enzyme-Linked Immunosorbent Assay
NTA	Nanoparticle Tracking Analysis
DLS	Dynamic Light Scattering
TEM	Transmission Electron Microscopy
SEM	Scanning Electron Microscopy
ARF6	ADP-Ribosylation Factor 6
SDS-PAGE	Sodium Dodecyl Sulfate Polyacrylamide Gel Electrophoresis
PBP	Polymer-based Precipitation
CA125	Cancer Antigen 125
TME	Tumor Microenvironment
TP53	Tumor Protein p53
CRC	Colorectal Cancer
HGS	Hepatocyte Growth Factor-Regulated Tyrosine Kinase Substrate
CSF	Cerebrospinal Fluid
PD	Parkinson’s Disease
EDIL3	Epidermal Growth Factor-Like Repeats and Discoidin I-Like Domains 3
EGFR	Epidermal Growth Factor Receptor
FAK	Focal Adhesion Kinase
CT	Computed Tomography
MRI	Magnetic Resonance Imaging
CA15-3	Carcinoma Antigen 15-3
HNSCC	Head and Neck Squamous Cell Carcinoma
TGF-β3	Transforming Growth Factor-beta 3
CRT	Chemoradiation Therapy
GWAS	Genome-Wide Association Studies
UBC	Urinary Bladder Cancer
HER2	Human Epidermal Growth Factor Receptor 2
TAAs	Tumor-Associated Antigens
TSAs	Tumor-Specific Antigens
MHC	Major Histocompatibility Complex
TCRs	T Cell Receptors
CD8+ T cells	Cytotoxic T Cells
Th cells	CD4+ Helper T Cells
APCs	Antigen Presenting Cells
DCs	Dendritic Cells
BCR	B Cell Receptor
MDSCs	Myeloid-Derived Suppressor Cells
TAMs	Tumor-Associated Macrophages
Tregs	Regulatory CD4+ T Cells
NK	Natural Killer
M-MDSCs	Monocytic MDSCs
PMN-MDSCs	Polymorphonuclear MDSCs
STAT	Signal Transducer and Activator of Transcription
ER	Endoplasmic Reticulum
NF-κB	Nuclear Factor Kappa B
cAMP	Cyclic Adenosine Monophosphate
IFN	Interferon
PTGES	Prostaglandin E Synthase
COX-2	Cyclooxygenase-2
C/EBPβ	CCAAT-Enhancer-Binding Protein-β
ARG1	Arginase 1
iNOS	Inducible Nitric Oxide Synthase
ROS	Reactive Oxygen Species
VEGF	Vascular Endothelial Growth Factor
IL	Interleukin
PGE2	Prostaglandin E2
PD-L1	Programmed Cell Death-Ligand 1
CCL18	Chemokine (C-C motif) Ligand 18
ECM	Extracellular Matrix
Teffs	Effector T Cells
CTLA-4	Cytotoxic T-Lymphocyte-Associated Protein 4
PD-1	Programmed Cell Death Protein 1
IFN-γ	Interferon-Gamma
PI3K	Phosphatidylinositol 3-Kinase
RAS	Rat Sarcoma Virus
CTL	Cytotoxic T Lymphocyte
Akt	Serine/Threonine Protein Kinase
TNF-α	Tumor Necrosis Factor
NSCLC	Non-Small Cell Lung Cancer
miRNA	MicroRNA
LNP	Lipid Nanoparticles
Th1	T-helper Cell Type 1
TRP2	Tyrosinase-Related Protein 2
IMC	Immature Myeloid Cells
SIRPα	Signal Regulatory Protein Alpha
circUHRF1	Circular Ubiquitin-Like with PHD and Ring Finger Domain 1 RNA
RGD	Arginine-Glycine-Aspartic Acid
MLKL	Mixed Lineage Kinase Domain-Like Protein
RIPK3	Receptor-Interacting Protein Kinase 3
GzmB	Granzyme B
FasL	Fas Ligand
TIM-3	T Cell Immunoglobulin and Mucin Domain 3
LAG-3	Lymphocyte Activation Gene 3
